# Hidradenitis Suppurativa: Dermatopathological Insights and Surgical Success Strategies

**DOI:** 10.1111/srt.70069

**Published:** 2024-09-19

**Authors:** Mahfujul Z. Haque, Frass Ahmed, Zachary Jodoin

**Affiliations:** ^1^ Department of Medicine Michigan State University College of Human Medicine Grand Rapids Michigan USA; ^2^ Department of Orthopedic Surgery UT Health San Antonio San Antonio Texas USA

Hidradenitis suppurativa (HS) is a debilitating skin condition with an estimated prevalence of up to 4% [1, 2, 3]. HS typically manifests in intertriginous areas and features painful abscesses, scars, and nodules [1, 2, 4]. Its systemic nature and the presence of comorbidities, such as obesity and diabetes, can complicate surgical healing [5]. HS has a strong recurrence rate as high as 35% following surgical closures, underscoring challenges in postoperative management [6]. Patients with HS more commonly experience wound dehiscence and surgical site infections, including osteomyelitis following surgical procedures such as total knee or hip arthroplasty [1]. HS has a global diagnostic delay of approximately 7.2 years, likely due to the lack of awareness among physicians [7]. Currently, there is a lack of literature addressing outcomes for patients with HS undergoing surgery. Surgeons need to be aware of exacerbating factors of HS and provide informed guidance to optimize patient health outcomes. This article outlines crucial considerations when treating patients with HS and provides evidence‐based recommendations for treating HS patients.

Perioperatively, patients require counseling about potential surgical complications, given HS's recurrent and inflammatory nature [2]. Surgeons must also focus on providing accessible resources for patient education. The HS Foundation website serves as a comprehensive resource for patients seeking information about HS and specialized HS care clinics [8]. Table 1 highlights certain HS recommendations for patients and provides a detailed list of HS specialty clinics across the United States.

**TABLE 1:** Recommendations for surgeons when treating patients with HS.

Circumstance	Recommendations	Resources
Pre‐operative care: Knowledge of HS and specialty clinic	Recommend visiting the HS Foundation website. Recommend attending HS conferences, such as the *Annual Symposium on Hidradenitis Suppurativa Advances*. Provide referral to HS specialty clinic. Recommend websites such as the HS Foundation to find nearby HS specialty clinics.	HS Foundation (https://www.hs‐foundation.org/) HS articles and blogs (https://www.hsconnect.org/articles HS specialty clinics (https://www.hs‐foundation.org/hs‐specialty‐clinics)
Pre‐operative care: Smoking	Recommend smoking cessation and use of nicotine replacement therapy techniques such as nicotine gum, patches, or lozenges. Recommend replacing time typically spent smoking with engaging in exercise, family, or healthy activities with friends.	Smoking reduction interventions (https://www.ncbi.nlm.nih.gov/pmc/articles/PMC6953262/) Quitting smoking (https://www.cdc.gov/tobacco/campaign/tips/quit‐smoking/index.html)
Pre‐operative care: Nutrition and diet	Recommend limiting aggravating foods such as dairy, sweets, red and processed meats, and refined carbohydrates. Recommend consuming alleviating foods such as vegetables, fruits, chicken, seafood, and nuts.	Nutrition, elimination diets, and HS (https://www.hsconnect.org/post/nutrition‐elimination‐diets‐and‐hs) HS diet (https://www.healthline.com/health/hidradenitis‐suppurativa‐diet)
Pre‐operative care: Physical activity	Recommend breathable or loose‐fitting clothing to limit skin friction. Recommend the use of moisture‐wicking clothing to limit sweat accumulation. Recommend other forms of physical activity such as swimming that may be more tolerable. Recommend immediately cleaning perspiration by showering or baby wipes	Exercise tips for patients with HS https://www.healthcentral.com/article/exercise‐with‐hidradenitis‐suppurativa Overview of exercising with HS https://hsdisease.com/exercise How to exercise with HS https://hsdisease.com/living/dont‐sweat
Pre‐operative care: Managing comorbidities	Counsel on managing metabolic syndrome and diabetes mellitus due to their exacerbation of HS symptoms and adverse impact on surgical outcomes. Advise on regular health monitoring and adherence to prescribed medications. Recommend lifestyle modifications, such as a balanced diet and regular physical activity, to mitigate comorbidities' effects.	
Perioperative care—Surgical site management strategies	Utilize non‐adherent dressings to decrease skin irritation. Apply negative pressure wound therapy for complex wounds. Consider silver‐impregnated dressings for their antimicrobial properties. Practice gentle handling of skin and subcutaneous tissue to minimize trauma. Execute surgical procedures that may involve wide excision and reconstruction with skin grafts or flaps, avoiding active or scarred HS lesions when planning the surgical site.	
Postoperative care—pain control	Develop a balanced pain management plan to address both surgical and HS‐related pain. Consider pharmacological therapy with agents such as adalimumab, especially for moderate to severe HS. Explore the use of biologic agents targeting IL‐17 and IL‐12/IL‐23 for their potential in pain management. Educate patients on pain management techniques, including the use of heat or cold therapy and relaxation techniques.	
Postoperative care—Collaboration with dermatologists	Engage in ongoing collaboration with dermatology specialists for continuous HS management. Follow dermatological guidance on the use of topical or systemic treatments to control HS inflammation. Consult dermatologists for advice on postoperative skin care routines compatible with wound care. Conduct regular dermatological assessments to promptly address HS exacerbations or complications.	

Abbreviation: HS, Hidradenitis suppurativa.

Effective management of HS is crucial to minimize postoperative complications in surgery. HS can significantly increase the risk of infection and impede wound healing, particularly in joint replacement surgeries [1]. This increased risk necessitates a comprehensive approach to the management and counseling of HS patients. A thorough preoperative assessment should be conducted to identify active HS lesions near surgical sites, as these lesions can be focal points for infection [9].

Before any procedure, HS can be controlled preoperatively through counseling, including advising patients on smoking cessation and dietary changes. The strong association of smoking with increased HS severity and flare‐ups makes smoking cessation a key recommendation [2]. Additionally, dietary guidance should be provided, focusing on avoiding foods that trigger HS flare‐ups, particularly after surgery [2, 4]. Foods such as red meat, dairy, sweets, and refined carbohydrates should be limited, while a diet rich in vegetables, fruits, chicken, seafood, and nuts, known to alleviate HS symptoms, is encouraged [2, 4]. This dual approach of smoking cessation and dietary modification not only reduces the risk of postoperative infections but also enhances wound healing.

Another aspect of perioperative management is addressing exercise‐induced pain caused by skin friction, a common issue in HS patients [10]. To minimize friction and improve comfort, patients are advised to wear loose‐fitting, breathable fabrics [10]. Additionally, moisture‐wicking fabrics are recommended to reduce sweat accumulation and associated symptom exacerbation [10]. This is particularly important as discomfort at the surgical site during exercise may be mistakenly attributed to the procedure itself, underscoring the need to control HS in conjunction with evaluating for surgical complications.

The effective management of comorbidities such as metabolic syndrome and diabetes mellitus is essential, as these conditions can exacerbate HS symptoms and adversely impact surgical outcomes [2]. Metabolic syndrome's contribution to systemic inflammation and diabetes's role in complicating wound healing are particularly relevant in the context of surgery. Patients should receive counseling on regular health monitoring, adherence to prescribed medications, and lifestyle modifications such as a balanced diet and regular physical activity [2].

The chronic and relapsing nature of HS necessitates a comprehensive approach to surgical management [2]. For surgical site management, the literature supports several strategies tailored to HS patients. These include using non‐adherent dressings to reduce skin irritation, employing negative pressure wound therapy for complex or non‐healing wounds, and considering advanced modalities like silver‐impregnated dressings for their antimicrobial properties [11, 12, 13]. Surgeons should also consider gentle handling of the skin and subcutaneous tissue during surgery to minimize trauma to affected areas [14]. Surgical procedures for HS often involve wide excision and reconstruction with skin grafts or flaps, particularly when primary closure is not feasible [15]. Surgeons must be vigilant in their surgical site planning, avoiding areas with active or scarring HS lesions when possible.

Postoperative care should focus on wound monitoring and effective pain control. HS itself can be a source of significant pain, which may be exacerbated post‐surgery [6, 9]. Therefore, a balanced pain management plan that addresses both surgical and HS‐related pain is essential. Pharmacological therapy, such as adalimumab, has been used in conjunction with surgery for moderate to severe HS [16, 17]. Additionally, the use of biologic agents targeting IL‐17 and IL‐12/IL‐23 has shown varying efficacy in treating HS, indicating the potential role of these agents in pain management for HS patients recovering from procedures [18]. Patient education on pain management techniques, including the use of heat or cold therapy and relaxation techniques, can be beneficial for preventing flare‐ups [19]. By adequately controlling pain, patients are more likely to participate in necessary rehabilitation exercises, thereby enhancing recovery and improving overall outcomes.

The postoperative phase for patients with HS undergoing surgery demands ongoing collaboration with dermatology specialists [3]. This interdisciplinary approach ensures continuous management of HS, which is crucial for preventing flare‐ups that can complicate surgical recovery. Dermatologists can provide guidance on the use of topical or systemic treatments to control HS inflammation and prevent new lesion formation that is compatible with postoperative healing [3]. Regular dermatological assessments can help in promptly identifying and addressing any HS exacerbations or complications. Collaborative care not only aids in the effective management of HS but also supports the overall healing process, contributing to successful surgical outcomes.

HS when coupled with surgical procedures, presents unique challenges due to its increased risk of infection, complications in wound healing, and the complex interplay with comorbidities. Thorough preoperative assessments, lifestyle modifications, and careful surgical site management are pivotal in minimizing postoperative complications. Addressing exercise‐induced discomfort, effective pain management, and the critical role of dermatological collaboration can enhance patient care. These insights offer a pathway for surgeons to provide effective care, contributing significantly to the holistic well‐being and recovery of this patient population.

## Ethics Statement

This study analyzed publicly available, anonymized internet data. No primary data collection involving human participants was conducted, and therefore, no ethical approval or consent procedures were required.

## Conflicts of Interest

The authors declare no conflicts of interest.

## Data Availability

The data that support the findings of this study are available from the corresponding author upon reasonable request.
